# Walnut Jug r 1 is Responsible for Primary Sensitization among Patients Suffering Walnut-Hazelnut 2S Albumin Cross-Reactivity

**DOI:** 10.1021/acs.jafc.4c03603

**Published:** 2024-07-31

**Authors:** Estela S. Castromil-Benito, Diana Betancor, Jorge Parrón-Ballesteros, Cristina Bueno-Díaz, Gloria Gutiérrez-Díaz, Javier Turnay, Manuel de las Heras, Javier Cuesta-Herranz, Mayte Villalba, Carlos Pastor-Vargas

**Affiliations:** † Department of Biochemistry and Molecular Biology, Faculty of Chemical Sciences, Complutense University of Madrid, Madrid 28040, Spain; ‡ Department of Allergy and Immunology, IIS-Fundación Jiménez Díaz UAM, Madrid 28015, Spain; § Red de asma, reacciones adversas y alérgicas (ARADyAL) RD16/0006/0013, Instituto de Salud Carlos III, Madrid 28029, Spain; ∥ RICORS Red de Enfermedades Inflamatorias (REI) - RD21/0002/0028, Instituto de Salud Carlos III, Madrid 28029, Spain

**Keywords:** food allergy, tree nut allergy, component-resolved diagnosis, allergen, seed storage proteins, 2S albumins, Jug r 1, Cor a 14, cross-reactivity, molecular diagnosis, primary sensitizer

## Abstract

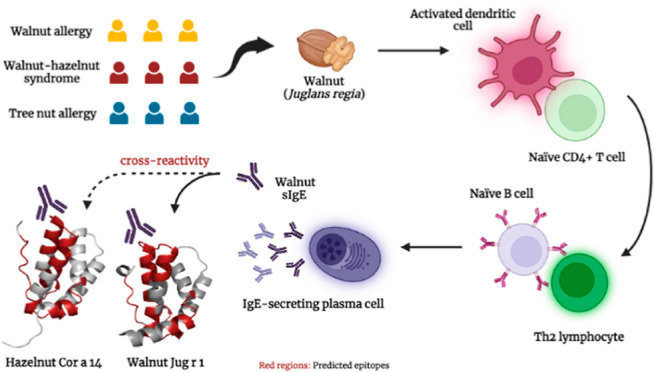

Walnut and hazelnut coallergy is a frequent manifestation in clinical practice whose molecular basis remains unclear. For this purpose, walnut-hazelnut cross-reactivity was evaluated in 20 patients allergic to one or both tree nuts and sensitized to their 2S albumins. Immunoblotting assays showed that 85% of patients recognized Jug r 1, walnut 2S albumin, which was associated with the development of severe symptoms; 50% of them corecognized hazelnut 2S albumin, Cor a 14. Both allergens were isolated using chromatographic techniques. Inhibition ELISAs revealed that Jug r 1 strongly inhibited the binding of Cor a 14-specific IgE, but Cor a 14 only partially inhibited Jug r 1-specific IgE binding. Our results showed that patients sensitized to walnut/hazelnut 2S albumins were not a homogeneous population. There were patients sensitized to specific epitopes of walnut 2S albumins and patients sensitized to cross-reactive epitopes between walnut and hazelnut, with Jug r 1 being the primary sensitizer.

## 1 Introduction

Tree nuts are one of the most common foods causing acute allergic reactions worldwide (range 1–3%).^1,2^ The onset of tree nut allergy often occurs at an early age and persists throughout the patient’s life, being associated with the development of the most severe symptoms.^3^ Tree nut allergy is of increasing clinical interest and concern because of the ubiquitous presence of nuts in various everyday products,^4,5^ which may lead to accidental ingestion with potentially fatal consequences. Likewise, nuts are a beneficial source of antioxidants, vitamins, and nutrients, particularly important in early stages of child development,^6^ so that nut allergy is also associated with nutritional deficiencies in allergic children.^7^

The epidemiology and sensitization profiles of tree nut allergy are variable and depend on age, region, lifestyle, and dietary habits,^8,9^ which together with severity and cross-reactivity with different nuts, make clinical management of allergic patients difficult in clinical practice.^10^ Many members of allergenic families are homologous proteins that are often involved in cross-reactivity, including seed storage proteins (2S albumins, vicilins, and 11S globulins), the major allergens in the UK and USA, and lipid transfer proteins (LTPs), the major ones in Mediterranean regions, along with the PR-10 family and profilins.^11^ In a clinical context, describing cross-reactivity between purified allergens (natural or recombinant) will improve the development of algorithms to optimize patient management, both for avoidance diet and allergen-specific immunotherapy (ASIT).^12−14^

2S albumins are small proteins (10–15 kDa) that exhibit structural stability and high resistance to thermal denaturation and gastrointestinal digestion. These properties are directly related to severe and near-fatal allergic reactions in sensitized patients.^15,16^ Although cross-reactivity between 2S albumins has not been well explored, some interesting cases have been described, such as the case of pistachio Pis v 1 and cashew Ana o 3 (*Anacardiaceae* family, sequence identity: 70%)^12,17^ and walnut Jug r 1 and pecan Car i 1 (*Juglandaceae* family, sequence identity: 88%).^16,18^ In both cases, cross-reactivity is based on the high degree of sequence identity between the 2S albumins. Nevertheless, this characteristic is also exhibited in the case of walnut and hazelnut 2S albumins, although both tree nuts do not belong to the same phylogenic family (*Juglandaceae* and *Betulaceae*, respectively).^16^ Moreover, walnut and hazelnut allergies have been strongly associated in clinical practice, suggesting a possible corecognition of homologous allergens which might involve these 2S albumins,^19^ as well as 11S globulin^19,20^ and/or 7S vicilin,^21^ according to the available information. The characterization of allergic syndromes attributed to different protein families is a goal for allergologists, as it will allow us to predict the severity of allergic reactions to related or unrelated foods, improve the outcomes of ASIT and promote the development of biosensors capable of detecting traces of allergens to prevent accidental ingestion.^22^

In accordance with this clinical association, this study has focused on the evaluation of walnut and hazelnut coallergy in patients sensitized to their 2S albumins (Jug r 1 and/or Cor a 14) using the natural allergens purified from tree nuts, thus preserving their native structure, in order to confirm the cross-reactivity between these proteins and, if so, to describe the primary sensitizer.

## 2 Materials and Methods

### 2.1 Population of Study

Patients diagnosed with primary allergy to walnut and/or hazelnut^23^ were selected from the Hospital Universitario Fundación Jiménez Díaz (Madrid, Spain). Inclusion criteria^23^ were a clinical history suggestive of food allergy, supported by a positive result in skin prick test (wheal diameter at least 3 mm larger than negative controls) with raw walnut (*Juglans regia*) and/or hazelnut (*Corylus avellana*) extract and significant values of specific IgE by ImmunoCap (≥0.35 kUA/L; ThermoFisher, Scientific, Uppsala, Sweden) to both sources and their respective 2S albumin, Jug r 1 and Cor a 14. Oral informed consent was obtained from all patients. The Hospital Universitario Fundación Jiménez Díaz Ethic Committee approved the study (protocol code PIC 201-20-FJD, approved on November 13th, 2020).

### 2.2 Walnut and Hazelnut Protein Extracts

Raw seeds of walnut and hazelnut were purchased from a local store in Madrid. Both nuts were crushed in liquid nitrogen and homogenized in extraction buffer (sodium borate 0.15 M, pH 8.0; 1 mM phenyl-methane-sulfonyl fluoride, PMSF) with magnetic stirring for 1 h at 4 °C. The suspension was centrifuged at 12,000*g* for 30 min at 4 °C and filtered three times before lyophilization. Then, the extract was delipidized with cold acetone for 30 min and centrifuged at 5500*g* for 15 min at 4 °C another three times. The organic phase was discarded, and the sediment was lyophilized, resuspended in 0.15 M ammonium bicarbonate buffer, pH 8.0, and stored at −20 °C until use. Protein concentration was determined by the Lowry method.^24^

### 2.3 SDS-PAGE and Western Blot Analysis

Extract quality and protein purity (2 and 20 μg/lane for purified proteins or extracts, respectively) were analyzed in 17% sodium dodecyl sulphate-polyacrylamide gel electrophoresis (SDS-PAGE) gels in the presence of 5% (v/v) β-mercaptoethanol if reducing conditions were required. Band densitometry was carried out with ImageLab software. For immunodetection of allergenic proteins, these were blotted onto Hybond ECL nitrocellulose membranes (Amersham Biosciences, Amersham, UK) using the Trans-Blot Semi Dry Transfer-Cell system (Bio-Rad, California, USA) in transfer buffer [48 mM Tris-base; 39 mM glycine; 0.0375% SDS (w/v); methanol 20% (v/v)]. Membranes were cut into 3 mm strips, blocked for 1 h at room temperature in blocking buffer [PBS-0.1% (v/v) Tween-20; 3% (w/v) skim milk powder], and incubated overnight at 4 °C under constant agitation with individual sera of the allergic patients (diluted 1/5 in blocking buffer). IgE binding was detected with a mouse antihuman IgE monoclonal IgG antibody (diluted 1/5000 in blocking buffer; Alk-Abelló, Madrid, Spain), followed by a peroxidase-labeled rabbit antimouse IgG polyclonal antibody (RAM-PO, 1/3000 dilution in blocking buffer; Pierce, Rockford, Illinois), both for 1 h at room temperature, with washes in PBS-0.1% (v/v) Tween-20 after each incubation. IgE binding was assessed by means of enhanced chemiluminescent signal using ECL substrates (Bio-Rad, California, USA), which was detected using a LAS-3000 CCD image analyzer (Fuji Photo Film Co., Ltd., Duluth, GA, USA) and processed with ImageJ software.

### 2.4 Isolation, Purification, and Identification of 2S Albumins

Seed storage proteins were isolated, as previously described by Bueno-Díaz et al.,^15^ by size exclusion chromatography using a Sephadex G-50 medium column (Sigma-Aldrich, St. Louis, Missouri, USA), equilibrated with 0.15 M ammonium bicarbonate, pH 8.0, at a flow rate of 3 mL/min. Low-molecular-mass protein batches (<30 kDa) were pooled and lyophilized. Fractions were resuspended in 20 mM ammonium bicarbonate, pH 8.0, and purified by reversed-phase high-performance liquid chromatography (RP-HPLC; Shimadzu, Kyoto, Japan), making use of a C18 Ultrasphere μbondpack column (Sigma-Aldrich, St. Louis, Missouri, USA) with an acetonitrile-0.1% (v/v) trifluoroacetic acid (TFA; Merck, Darmstadt, Germany) elution gradient from 0 to 80% in 50 min at a constant flow rate of 1.5 mL/min. The obtained batches were immediately lyophilized after elution and resuspended in 20 mM ammonium bicarbonate pH 8.0. Purified proteins were quantified by absorption spectroscopy at 280 nm using their theoretical molar extinction coefficient, estimated with the ExPASy ProtParam tool after removing the predicted signal peptide of the annotated sequence with Signal-P 5.0. In parallel, their immunological recognition was tested by Western blot. Identification of purified proteins was assessed by matrix-assisted laser desorption/ionization (MALDI)-time of flight (TOF) mass-spectrometry, in collaboration with the Proteomics Unit of the Complutense University of Madrid.

### 2.5 Analysis of Sequence Identity and Epitope Mapping

*In silico* sequence comparisons were performed to theoretically evaluate the cross-reactivity between Jug r 1 and Cor a 14. Signal peptide of annotated sequences (UniProt) was predicted and removed using the Signal-P 5.0 bioinformatic service (https://services.healthtech.dtu.dk/services/SignalP-5.0/), and sequence alignment and estimation of the percentage of similarity and sequence identity were carried out with the *EMBOSS Needle Pairwise Sequence Alignment* bioinformatic tool (https://www.ebi.ac.uk/Tools/psa/emboss_needle/). Predictions of B-cell linear epitopes were obtained with the *Bepipred Linear Epitope Prediction* 2.0 program (http://tools.immuneepitope.org/bcell/) and later aligned.

### 2.6 Cross-Reactivity Assays

IgE-mediated cross-reactivity was assessed by indirect and inhibition ELISA in 96-well polystyrene plates (Costar, Massachusetts, USA) coated overnight at 4 °C with purified protein (5 μg/mL in PBS, pH 7.6; 100 μL/weel). Plates were washed with PBS-0.05% (v/v) Tween-20 (PBS-T) after each incubation and blocked with 2% (w/v) BSA in PBS-T. For inhibition ELISA, each serum was preincubated with purified proteins with a control concentration (100 μg/mL serum), increasing amounts for the inhibition curves (0.001–100 μg/mL serum) or BSA as a negative control at room temperature for 4 h with constant agitation. Inhibition mixtures (diluted 1/10, 1/20, or 1/40 in blocking buffer according to the IgE level of the serum used) were added to the plate and kept at 37 °C for 2 h. Plates were then incubated with a mouse antihuman IgE monoclonal antibody (diluted 1/5000 in blocking buffer; ALK-Abelló, Madrid, Spain), followed by a peroxidase-labeled goat antimouse polyclonal IgG antibody (GAM-PO, 1/3000 dilution in blocking buffer; Dako, Glostrup, Denmark), both for 1 h at room temperature. IgE binding was detected using 3,3′,5,5′-tetramethylbenzidine (TMB, 100 μL/well) and a Multiskan FC microplate reader (ThermoFisher, Massachusetts, USA) for absorbance measurement at 650 nm. Nonspecific antibody binding was ruled out by subtracting the absorbance of the uncoated wells used as controls. Measurements of absorbance greater than three times the mean of nonatopic controls were considered positive, and the percentage inhibition was calculated according to the following formula: inhibition (%) = [1 – (OD_650_ with inhibitor/OD_650_ without inhibitor)] × 100.

### 2.7 Statistical Analysis

Qualitative variables were expressed as percentages and calculated with 95% confidence intervals. For quantitative variables, means and standard deviation of experimental duplicates were calculated, and for specific IgE results, medians and 25th (Q1) and 75th (Q3) percentiles were given. Values were considered significant at a *p*-value of less than 0.05. Statistical analysis was carried out using GraphPad InStat 6 software.

## 3 Results

### 3.1 Clinical Features of Allergic Patients

A total of 20 patients allergic to walnut and/or hazelnut were included in the study, whose clinical characteristics are summarized in Table 1. The mean age was 16.2 years (range 5–53 years) with a notable predominance of juniors (<18 years old; 16 patients, 80%) and male patients (15 patients, 75%). Allergic systemic reactions were observed in 16 patients (80%), 5 of them being anaphylactic ones. Only 4 patients (15%) presented mild symptoms, highlighting oral allergy syndrome (OAS). The median sIgE level to hazelnut extract was 3.2 kU/L (Q1-Q3:0.6-15.0) and to Cor a 14 was 1.6 kU/L (Q1-Q3:0.04-4.6) while sIgE level to walnut was 10.5 kU/L (Q1-Q3:6.1-37.5) and to Jug r 1 was 7.4 kU/L (Q1-Q3:3.8-16.9), which were three and five times higher, respectively. According to ImmunoCAP IgE level classification, 15% of the patients have similar IgE levels for both Jug r 1 and Cor a 14, while 50% have higher IgE levels to Jug r 1. The rest were monosensitized to walnut 2S albumin. Only three patients were allergic exclusively to walnut and/or hazelnut, whereas the rest of the patients were sensitized to one or more foods, including almond and pistachio (both 62%), cashew (69%), and peanut (44%).

**Table 1:** Clinical and Demographic Characteristics of Allergic Patients^a^

Patient N°	Sex/Age	Hazelnut IgE (kU/l)	rCor a 14 sIgE (kU/l)	Walnut IgE (kU/l)	rJug r 1 sIgE (kU/l)	Initial symptoms	Food involved
1	M/5	0.35		6.32	7.48	*Systemic*	*W*
2	F/5	0.23	0.19	5.64	2.99	*Local*	*H*, *W*
3	M/53	0.15		0.73	0.50	*Systemic*	*H*, *W*
4	M/10	2.47	4.17	10.50	14.6	*Systemic*	*H*
5	F/31	6.20	3.36	20.50	7.92	*Systemic*	*H*, *W*
6	F/8	2.76	0.51	7.22	4.7	*Systemic*	*W*
7	M/7	15.00	2.42	70.90	19.10	*Systemic*	*H*, *W*
8	M/5	3.22	3.75	10.50	8.71	*Systemic*	*H*
9	M/12	5.15	1.61	16.80	7.36	*Systemic* (*A*)	*W*
10	M/52	0.6		8,9	6.62	*Local*	*H*
11	M/14	50.60	11.9	65.9	35.90	*Systemic* (*A*)	*H*, *W*
12	M/12	24		78.7	>100	*Systemic* (*A*)	*W*
13	M/16	>100	37	>100	>100	*Systemic* (*A*)	*H*
14	F/26	45	57.90	>100	>100	*Systemic*	*H*, *W*
15	M/14	36.7	4.8	78.90	48	*Systemic*	*W*
16	F/10	1,27	1.61	10,5	14.6	*Local*	*W*
17	M/12	0.1		0.81	0.49	*Local*	*W*
18	M/13	5,15	1.37	16,8	7.36	*Systemic* (*A*)	*W*
19	M/8	4	9.83	27,7	16.7	*Systemic*	*W*
20	M/12	1.41	1.43	1.12	-	*Systemic*	*W*

a Abbreviations: A, anaphylaxis; F, female; H, hazelnut; M, male; W, walnut.

### 3.2 Allergen Pattern Recognition from Total Protein Extracts

**Figure 1:**
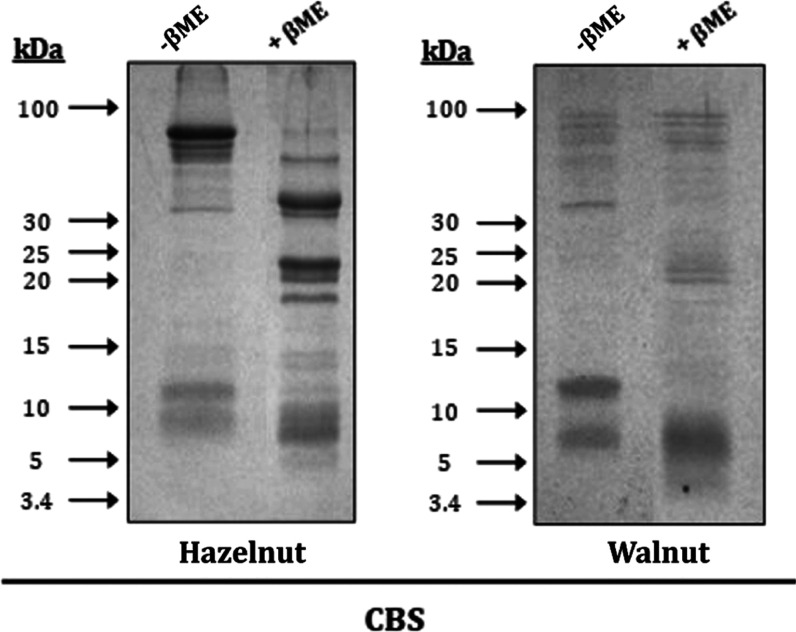
. Composition of total hazelnut and walnut protein extract determined by Coomassie blue staining (CBS) of 17% SDS-PAGE under reducing (+βME) and nonreducing conditions (−βME).

**Figure 2:**
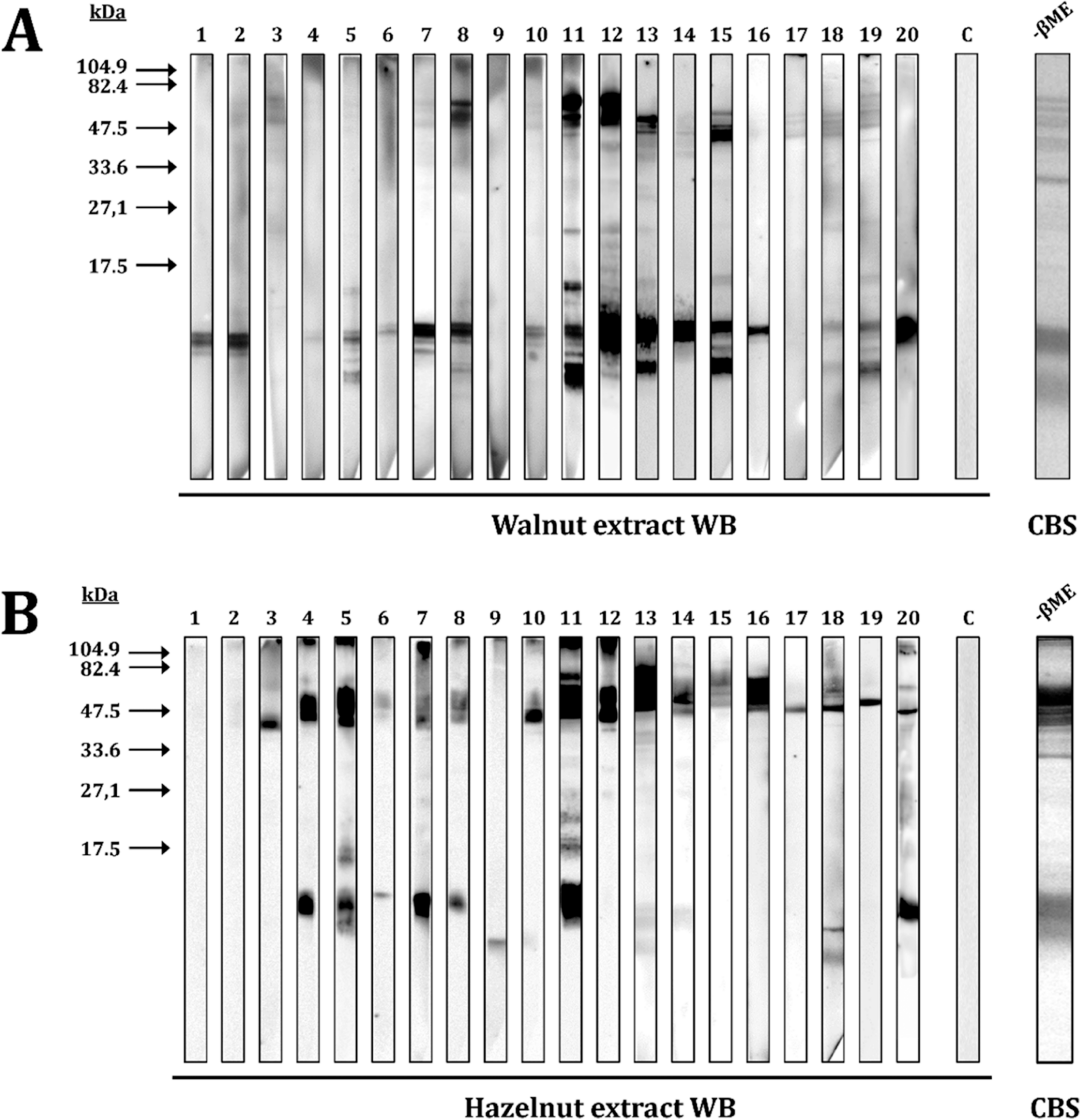
Western blot analysis of the individual allergic patient sera (numbered 1–20 according to Table 1) against (A) walnut extract or (B) hazelnut extract. C, nonatopic serum control; CBS, Coomassie blue staining of the respective extract under nonreducing conditions (−βME).

SDS-PAGE of walnut and hazelnut revealed multiple protein bands with apparent molecular masses ranging from 5 to 100 kDa (Figure 1). Immunoblotting assays of patients’ individual sera revealed a spectrum of IgE-reactive proteins (Figure 2), the ∼11 kDa one being the most frequently recognized (85 and 45% in walnut and hazelnut extract, respectively), which might correspond to 2S albumins due to the behavior of the protein in the presence of β-mercaptoethanol, splitting in two smaller polypeptide chains (∼8 and 4 kDa). High-molecular-mass bands, which could include 11S (48 kDa) and 7S globulins (40 kDa) among others, were especially recognized in hazelnut extract (75%) in contrast to the walnut one (35%), in which 2S albumins were found to be the major allergen. This result is consistent with the SDS-PAGE analysis (Figure 1), where it was estimated by densitometry that 2S albumin is found in a higher proportion in the walnut extract (30%) compared to the hazelnut one (10%). Finally, the 9 kDa band, whose molecular mass corresponds to an LTP, was recognized for 35 and 20% of patients in walnut and hazelnut extracts, respectively.

According to the allergen recognition pattern and the clinical history of the patients, sensitization to 2S albumins correlated with the development of severe symptoms in 92% and 80% of cases after ingestion of walnut and hazelnut, respectively. Sensitization to both proteins was also associated with a slight increase in anaphylactic reactions (30%).

By contrast, sensitization to high-molecular-mass proteins was present in 100% of the cases where mild symptoms developed, regardless of the food involved. In addition, the only patient with severe symptomatology who did not recognize Jug r 1 (patient #9) could suffer from an LTP-syndrome due to sensitization to a low-molecular-mass band (9 kDa) and a positive result to peach Pru p 3 on ImmunoCAP (2.64 kU/L).

Due to their high recognition rate and their correlation with the development of severe symptoms, 2S albumins were considered for *in silico* sequence comparison and *in vitro* cross-reactivity assays.

### 3.3 *In Silico* Analysis of Jug r 1 and Cor a 14 Primary Structures

**Figure 3:**
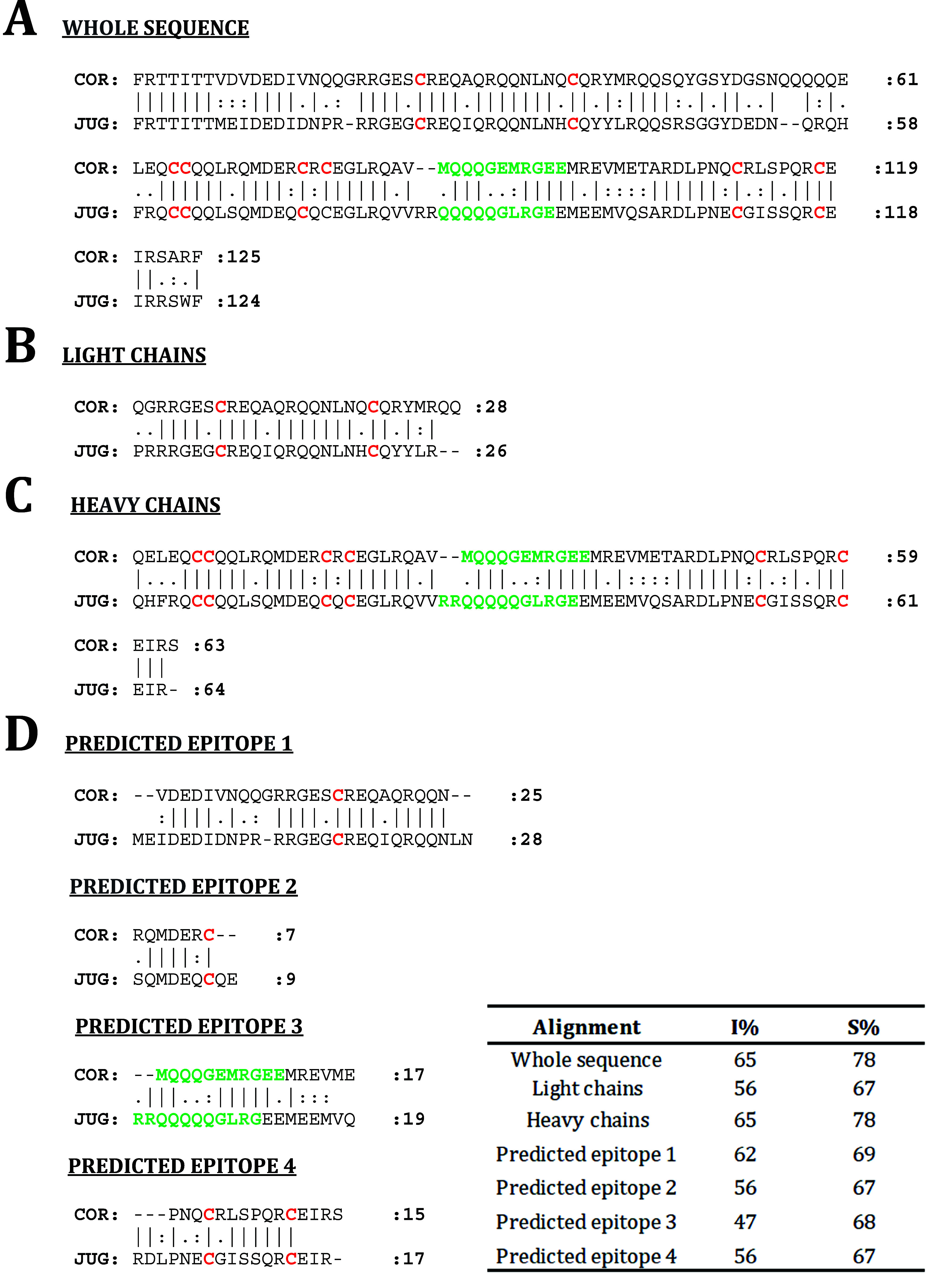
(A) Sequence alignment of Jug r 1 (JUG) and Cor a 14 (COR) amino acid sequence, (B) light chains, (C) heavy chains, and (D) predicted epitopes. “|” indicates amino acid conservation, “:” a conservative substitution, “.” a semiconservative substitution, and “-” a mismatch. Cysteine pattern is shown in red and hypervariable region in green. I %/S %: sequence identity percentage/sequence similarity percentage.

Sequence alignment was performed first with complete amino acid sequences (Figure 3A), revealing a sequence identity percentage (I %) of 65% and a sequence similarity percentage (S %) of 78%. For more precise results, it was considered to carry out the alignment with the light- (Figure 3B) and heavy-chain amino acid sequences (Figure 3C) separately, using those annotated according to the related literature.^15,25−27^ In this way, heavy-chain alignment showed the highest I %, up to 65% as in the case of the whole sequence. Four linear epitopes have been predicted within the sequence of both proteins using *in silico* techniques; three of them correspond to the heavy chain and one to the light one. These results are consistent with the described data for other allergenic 2S albumins in which there is a greater recognition of the heavy chain or is even exclusive.^15,28^ Moreover, the alignment of those epitopes had similar S % values (range 47–62%; Figure 3D), suggesting that possible linear epitopes from the two proteins could lead to a cross-reactivity process, with the epitopes containing the hypervariable region, known to include the main immunogenic IgE-epitopes in 2S albumins, the ones with fewer I % values (Figure 3D).

### 3.4 *In Vitro* Cross-Reactivity Analysis between Jug r 1 and Cor a 14

**Figure 4:**
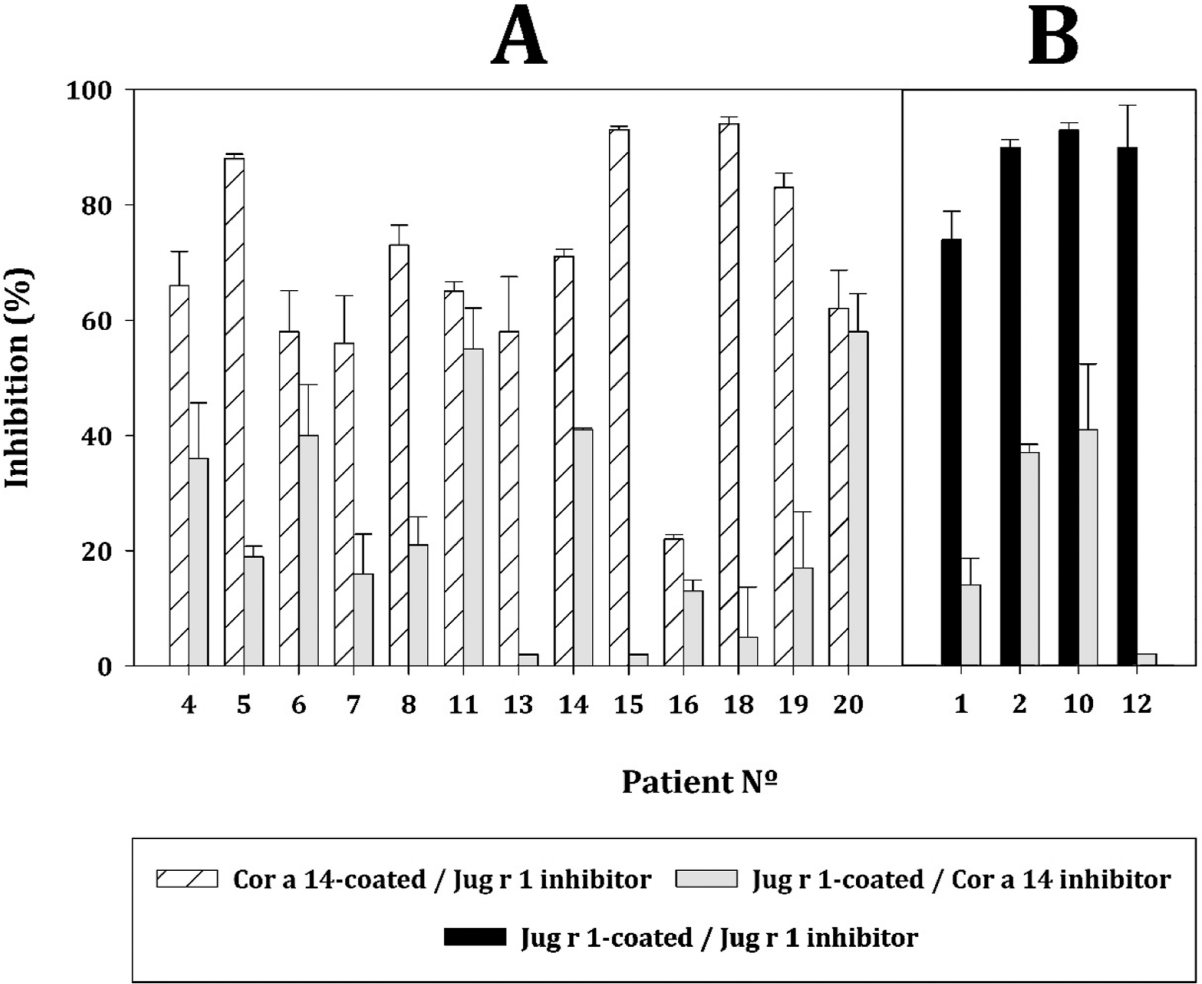
Inhibition percentages of purified protein recognition. (A) Double-sensitized patient sera. (B) Jug r 1 monosensitized patient sera. White bars: Plate coated with Cor a 14, sera inhibited with Jug r 1; gray bars: plate coated with Jug r 1, sera inhibited with Cor a 14; black bars: plate coated with and sera were inhibited with Jug r 1. Serum used is indicated in the *X*-axis as “Patient N°” according to the Table 1.

Inhibition ELISA was performed with solid-phase purified Jug r 1 and Cor a 14 using the 13 individual sera of double walnut/hazelnut sensitized patients and the four Jug r 1 monosensitized patient sera, according to ImmunoCAP, preincubated with both allergens (Figure 4). In the case of double-sensitized patients, Jug r 1 was always able to inhibit IgE binding to Cor a 14 in all cases, reaching an average inhibition rate of 70% in the assayed conditions, reaching almost total inhibition in four cases (∼25%). However, in no case was Cor a 14 able to inhibit IgE binding to Jug r 1 completely, reaching an average inhibition rate lower than 25%. Surprisingly, in Jug r 1 monosensitized patients, according to Western Blot and ImmunoCAP, Cor a 14 was able to inhibit Jug r 1 sIgE binding, although not in a comparable percentage.

**Figure 5:**
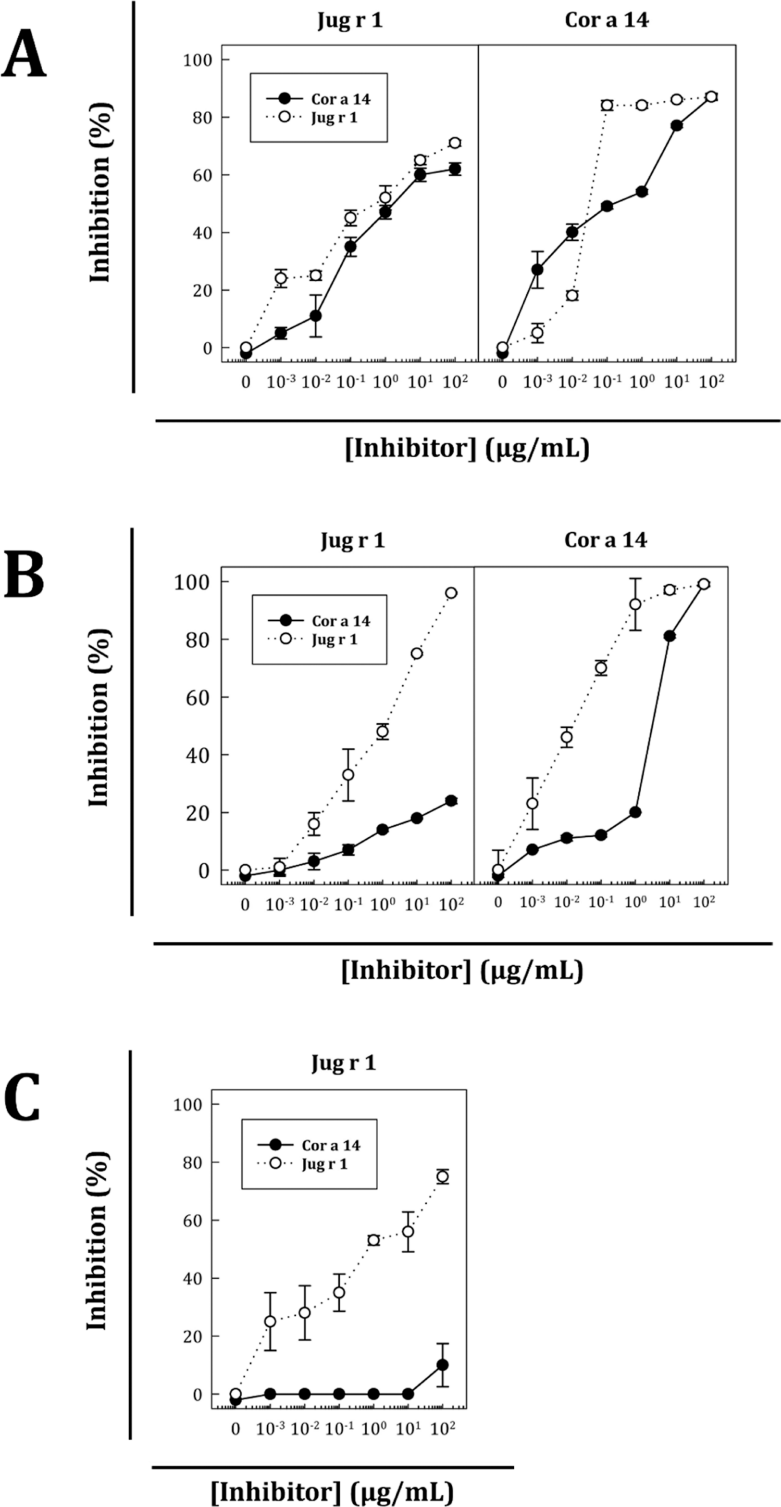
Inhibition curves of purified protein recognition, Jug r 1 or Cor a 14, as indicated in each upper axis. (A) Double-sensitized patient serum #11. (B) Double-sensitized patient serum #14. (C) Jug r 1 monosensitized patient serum #12. Sera was inhibited with increasing amounts of Cor a 14 (⧳) or Jug r 1 (Φ).

To confirm Jug r 1 as the primary sensitizer, increased quantities of purified protein were used to perform inhibition ELISA with three selected patients with high sIgE values to increase the sensitivity of the titration: two patients double-sensitized to Jug r 1 and Cor a 14 who differed in IgE levels against walnut and hazelnut extracts (patients #11 and #14, Figure 5A,B) and one sensitized only to Jug r 1 as an internal control (patient #12, Figure 5C). In both double-sensitized patients, Jug r 1 achieved more than 90% inhibition of Cor a 14 sIgE binding and required less concentration to reach high inhibition values. By contrast, in patient #11, Cor a 14 inhibited the binding of Jug r 1 sIgE with a similar percentage as Jug r 1 itself (Figure 5A), although it did not exceed 60% inhibition, while in patient #14 (Figure 5B), Cor a 14 could not reach a significant inhibition (less than 20%). In the case of the monosensitized patient sera, Cor a 14 did not inhibit Jug r 1 sIgE binding even at the higher dose.

## 4 Discussion

In this report, we evaluated the cross-reactivity, a complex and not well-established process, between two 2S albumins from different phylogenic origins, walnut Jug r 1 and hazelnut Cor a 14, using the purified natural allergens from both tree nuts, which are some of the main sources of allergy in Spain.

Sensitization to 2S albumins is frequent in juniors, and it has been associated with severe allergic reactions,^15,16,29^ which is consistent with the allergic profiles of our cohort of study. Results also confirmed the existence of this IgE cross-reactivity reaction between both proteins across common epitopes but also revealed the presence of Jug r 1-specific epitopes, which might be responsible for walnut 2S albumin monosensitization, a fact that may also be related to its abundance in the walnut extract compared to the hazelnut one. On one hand, these data deserve to be highlighted as important and relevant in the management of tree nut-allergic patients, since in cases of patients with walnut and hazelnut allergy due to sensitization to 2S albumins, walnut Jug r 1 tolerance induction treatment should result in tolerance to both allergens, as shown by some preliminary studies of desensitization with whole walnut extract.^13^ On the other hand, by analogy to cashew-pistachio syndrome, walnut-hazelnut syndrome would define patients sensitized exclusively to their 2S albumins, exhibiting cross-reactivity between them with negative skin prick test and absence of sIgE to other tree nuts. Therefore, patients suffering from this syndrome would have a low risk of developing symptoms by eating tree nuts other than walnuts and hazelnuts.

Villalta et al.^19^ observed a cross-reactivity process between different seed storage proteins by inhibition ImmunoCAP in a short series of 13 patients with walnut or hazelnut primary allergy using whole walnut extract. In 2021, Bueno-Díaz et al.^15^ described a recognition cluster restricted to hazelnut and walnut 2S albumins using a pool of sera from hazelnut-allergic patients.

In our study, cross-reactivity between both natural 2S albumins was demonstrated by inhibition ELISA assay using two different patient profiles: the first one sensitized to both Jug r 1 and Cor a 14 and the second one just to Jug r 1. No studies could be performed in patients only sensitized to Cor a 14 due to the absence of this profile in our cohort of study. Results showed that Jug r 1 strongly blocks the binding of Cor a 14 sIgE in almost all cases, whereas Cor a 14 only inhibits partially the binding of Jug r 1 sIgE.

According to the inhibition curves, two phenotypes of patients can be distinguished: a case of cross-reactivity through common epitopes (Figure 5A), since Cor a 14 achieves the same percentage of inhibition as Jug r 1 when used both as inhibitors of Jug r 1 sIgE binding and a Jug r 1 primary sensitization through specific and common epitopes (Figure 5B). Specific epitopes in Jug r 1 explain why Jug r 1 completely inhibits Cor a 14 sIgE binding, but Cor a 14 does not inhibit Jug r 1 sIgE binding. In agreement with all above, all results point toward Jug r 1 as the primary sensitizer in walnut and hazelnut 2S albumins-sensitized patients.

2S albumins are major allergens widely distributed in different plant-derived food sources. These proteins are characterized by their small, heterodimeric, and compact structure that confers on them high stability and resistance to thermal denaturation and gastrointestinal digestion.^15,30,31^ They are also encoded by a multigene family, leading to numerous isoforms that undergo post-translational modifications like proteolytic digestion or glycosylation.^30,31^ Despite the relatively low amino acid sequence identity they exhibit, structural features of these proteins point to conformational epitopes that explain the cross-reactivity between them. Surprisingly, the sequence identity between walnut and hazelnut 2S albumins reaches a value of 65%, which is close to the threshold suggested by *Aalberse*(32) for considering a cross-reactivity process between allergens from different phylogenic families. Moreover, sequence alignment of the predicted epitopes evidence the potential cross-reactivity between these 2S albumins, not only at the conformational level but also at the sequence level. However, according to *in vitro* results, Jug r 1 appears to have unique specific epitopes that are absent in Cor a 14 since this protein did not completely inhibit Jug r 1 sIgE binding in any case.

The possible discrepancies between the IgE binding analyses in this population depend on the heterogeneity among the immunologic techniques used for the *in vitro* diagnostics and the structure of the protein used. ImmunoCAP included in this study, in contrast to ELISA, uses recombinant allergens, which in the case of 2S albumins may not have a comparable tertiary structure as the natural, mainly due to the difficulties in producing in any heterologous system these heterodimeric proteins. For example, the only Cor a 14 monosensitized patient according to ImmunoCAP as shown in Table 1 (patient #20) turned out to be positive for Jug r 1 recognition in both Western Blot and ELISA assays from this work, which is the reason why this patient was eventually included in the double-sensitized group. Similarly, patient #9 was excluded from the analysis, despite being sensitized to both 2S albumins according to ImmunoCAP, because no reactivity was observed when assayed in both Western Blot and ELISA. On the other hand, Western Blot differs from the other two techniques because immunodetection is performed on denatured proteins, so only partially structured epitopes can be considered. This is why patients #1, #2, #3, and #10, despite being monosensitized to Jug r 1 according to immunoblotting (and also according to ImmunoCAP; Table 1 and Figure 1), showed inhibition of Jug r 1 sIgE binding using Cor a 14 as an inhibitor in ELISA, where conformational epitopes are kept intact because proteins are assayed in their native structure.

In conclusion, the results of this study show that patients sensitized to walnut and hazelnut 2S albumin are not a homogeneous population, in which patients can be found to be sensitized to specific epitopes of walnut 2S albumin and, to a greater extent, patients sensitized to cross-reactive epitopes between walnut and hazelnut 2S albumins (including walnut-hazelnut syndrome). In the last case, the primary sensitizer would be walnut Jug r 1. These findings will not only facilitate the management of patients in clinical practice but also guide their treatment by proposing Jug r 1 as a candidate for immunotherapy in the patient phenotypes characterized. Likewise, future studies should be conducted to determine the true extent of these results in larger patient cohorts, as well as in different geographic and climatic areas.

## Notes

All authors have participated in the design and interpretation of data for the work and revising critically the article for important intellectual content. All have approved the submission of this manuscript.

## Abbreviations

ASIT: antigen-specific immunotherapy
β-ME: β-mercaptoethanol
GAM: goat antimouse
HPLC: high-pressure liquid chromatography
LTP: lipid transfer protein
MALDI-TOF: matrix-assisted laser desorption/ionization-time of flight
OAS: oral allergy syndrome
PBS-T: phosphate buffered saline—Tween 20
PMSF: phenylmethylsulfonyl fluoride
PO: peroxidase
RAM: rabbit antimouse
IgE: specific-Immunoglobulin E
TFA: trifluoroacetic acid
TMB: 3,3′,5,5′-tetramethylbenzidine
